# Iron accumulation in the choroid plexus, ependymal cells and CNS parenchyma in a rat strain with low‐grade haemolysis of fragile macrocytic red blood cells

**DOI:** 10.1111/bpa.12920

**Published:** 2021-01-29

**Authors:** Isabella Wimmer, Cornelia Scharler, Taro Kadowaki, Sophie Hillebrand, Barbara Scheiber‐Mojdehkar, Shuichi Ueda, Monika Bradl, Thomas Berger, Hans Lassmann, Simon Hametner

**Affiliations:** ^1^ Department of Neurology Medical University of Vienna Vienna Austria; ^2^ Department of Neuroimmunology Center for Brain Research Medical University of Vienna Vienna Austria; ^3^ Department of Neurology Dokkyo Medical University Tochigi Japan; ^4^ Department of Medical Chemistry and Pathobiochemistry Medical University of Vienna Vienna Austria; ^5^ Department of Histology and Neurobiology Dokkyo Medical University Tochigi Japan; ^6^ Division of Neuropathology and Neurochemistry Department of Neurology Medical University of Vienna Vienna Austria

**Keywords:** cerebrospinal fluid, erythrocyte osmotic fragility, iron overload, kidney proximal tubule, multiple sclerosis, zitter rat

## Abstract

Iron accumulation in the CNS is associated with many neurological diseases via amplification of inflammation and neurodegeneration. However, experimental studies on iron overload are challenging, since rodents hardly accumulate brain iron in contrast to humans. Here, we studied LEWzizi rats, which present with elevated CNS iron loads, aiming to characterise choroid plexus, ependymal, CSF and CNS parenchymal iron loads in conjunction with altered blood iron parameters and, thus, signifying non‐classical entry sites for iron into the CNS. Non‐haem iron in formalin‐fixed paraffin‐embedded tissue was detected via DAB‐enhanced Turnbull Blue stainings. CSF iron levels were determined via atomic absorption spectroscopy. Ferroportin and aquaporin‐1 expression was visualised using immunohistochemistry. The analysis of red blood cell indices and serum/plasma parameters was based on automated measurements; the fragility of red blood cells was manually determined by the osmotic challenge. Compared with wild‐type animals, LEWzizi rats showed strongly increased iron accumulation in choroid plexus epithelial cells as well as in ependymal cells of the ventricle lining. Concurrently, red blood cell macrocytosis, low‐grade haemolysis and significant haemoglobin liberation from red blood cells were apparent in the peripheral blood of LEWzizi rats. Interestingly, elevated iron accumulation was also evident in kidney proximal tubules, which share similarities with the blood–CSF barrier. Our data underscore the importance of iron gateways into the CNS other than the classical route across microvessels in the CNS parenchyma. Our findings of pronounced choroid plexus iron overload in conjunction with peripheral iron overload and increased RBC fragility in LEWzizi rats may be seminal for future studies of human diseases, in which similar constellations are found.

## Graphical abstract

LEWzizi rats present with pronounced choroid plexus iron overload in conjunction with elevated levels of iron and cell‐free haemoglobin in the blood and increased RBC fragility. In several human diseases of diverse aetiologies, a pronounced accumulation of iron in choroid plexuses has already been described. Our study highlights the importance of choroid plexuses as iron entry sites into the CNS and their linkage with peripheral iron loads and alterations in red blood cell biology.

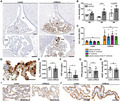

## 1 INTRODUCTION

Iron is an essential co‐factor for vital metabolic functions, like DNA and lipid synthesis or oxidative phosphorylation (1, 2, 3). Due to its high pro‐oxidative capacity, the import, storage, export and transport of iron must be tightly regulated (4). Iron that is not used for metabolic processes is, therefore, kept in a redox‐inactive state by either (i) storage in cytoplasmic ferritin shells or (ii) binding to proteins (e.g. transferrin, ferritin) and low molecular weight molecules in the extracellular environment (5). In the CNS, myelination and myelin maintenance particularly depend on iron. Accordingly, the majority of iron in the healthy CNS is stored in oligodendrocytes and myelin (3, 6). Under normal conditions, iron accumulates in the CNS of mammals with increasing age without major toxic effects (7, 8, 9). The degree of iron accumulation varies greatly between brain regions, with the nuclei of the deep grey matter becoming particularly iron‐rich (8, 10). Iron dysregulation in the CNS, i.e. both accumulation and deficiency, can have harmful effects on brain tissue. Abnormal iron accumulation relates to acquired (e.g. Parkinson's disease, Alzheimer's disease) and congenital neurodegenerative diseases (e.g. Friedreich's ataxia, pantothenate kinase‐associated neurodegeneration, neuroferritinopathies, aceruloplasminemia) (11, 12, 13, 14, 15, 16). In contrast, iron deficiency in the CNS of infants leads to developmental delay and cognitive problems (17, 18).

Uncontrolled entry of iron from the blood into the CNS is prevented by vascular barrier systems, i.e. the blood–brain barrier, the blood–cerebrospinal fluid (CSF) barrier and the blood–leptomeningeal barrier (19). Iron import has traditionally been studied at its classical entry site, the endothelial blood–brain barrier, while data on non‐classical iron entry sites are sparse. As such, choroid plexuses are of central interest. They are densely vascularised and blood flow rates per volume are five times higher than in the brain parenchyma (20). Choroid plexus epithelial cells express molecules for iron import, storage and export (21). Alterations in the iron content of choroid plexuses have been observed for diverse human diseases such as β‐thalassemia, fragile X‐associated tremor/ataxia syndrome (FXTAS), or alcoholism (22, 23, 24, 25, 26). Also, upon repeated therapeutic transfusions of red blood cell (RBC) concentrates or in the genetic iron overload condition hemochromatosis, a “choroid plexus iron overload sign” was described in magnetic resonance imaging (27, 28). Thus, accumulation of iron in the choroid plexuses manifests in manifold diseases with diverse aetiologies underscoring the need to further expand the knowledge on iron imbalances at this site.

Experimental rodent studies on iron overload are limited by intrinsically low CNS iron levels in these models, which hardly increase with age (29). Rats harbouring a spontaneous mutation in the attractin (*Atrn*) gene (LEWzizi and zitter (zi/zi) rats) show abnormally high iron levels in the CNS parenchyma, as we and others have shown before (30, 31, 32). Using this rat model, we aimed at investigating changes in iron loading in the ventricular system including choroid plexus, CSF and ependyma. We, indeed, found a pronounced accumulation of iron in choroid plexus epithelial cells and ependymal cells of the ventricle linings. Concurrent with elevated CNS iron, iron as well as free haemoglobin levels in the LEWzizi plasma were significantly increased accompanied by RBC macrocytosis and significantly increased RBC fragility leading to low‐grade haemolysis. Our data underscore the importance of non‐classical iron entry sites into the CNS and their linkage with peripheral iron loads and alterations in RBC biology.

## 2 MATERIALS AND METHODS

### 2.1 Animals

LEWzizi (LEW.SD‐Atrn^zi/zi^) rats (32) derived from zitter (zi/zi) rats (Sprague Dawley outbreds) backcrossed on the Lewis background were housed in the Center for Biomedical Research (Medical University Vienna) under standardised conditions.

### 2.2 Automated blood analyses

Rats were sacrificed by an overdose of CO_2_ and blood was immediately drawn by heart puncture. K_3_EDTA blood was used for complete blood counts examined on an Avida 2120i machine (Siemens) and for reticulocyte counts examined by an external company (IN VITRO ‐ Labor für veterinärmedizinische Diagnostik und Hygiene GmbH). Plasma derived from lithium–heparin blood was used for the routine determination of haemoglobin, non‐haem iron, lactate dehydrogenase, haptoglobin and potassium levels on a Hitachi cobas c 311 analyser (Roche). Serum (coagulated for 1 h at room temperature; then centrifuged twice at 1500 × g for 10 min) was used for measurements of folate and vitamin B12 levels carried out by IN VITRO.

### 2.3 Osmotic fragility test

The applied method based on (33) downscaled to a 1.5 mL tube format. From a buffered sodium chloride stock solution, osmotically equivalent to 100 g/L of NaCl (1.54 M NaCl, 0.0962 M Na_2_HPO_4_, 0.015 M NaH_2_PO_4_), a series of hypotonic working solutions ranging from 0.1 to 0.8 g/L of NaCl were prepared. K_3_EDTA blood (20 µL) was mixed with 1 mL of each hypotonic solution and incubated at room temperature for 30 min. After centrifugation (1200 × g for 5 min), absorbances were measured at 560 nm (GloMax® Multi Detection System, Promega). The percentage of haemolysis was calculated using distilled water as a 100% haemolysis reference and plotted against respective NaCl concentrations. A sigmoidal 4PL curve fit was conducted and the IC50 (concentration of NaCl causing 50% haemolysis of RBCs) calculated using GraphPad Prism® v6.01.

### 2.4 CSF withdrawal

CSF was collected immediately after rat sacrifice (CO_2_) by puncture of the cisterna magna with a 27G needle attached to a 1 mL syringe via a long transparent tubing. Per animal, 100–200 µL CSF was obtained, subsequently centrifuged (1000 × g for 15 min at 4°C) and stored at −80°C.

### 2.5 ELISA

Rat albumin (ab108790; Abcam) and rat sCD163 (#MBS729231; MyBioScource) ELISA Kits were performed according to the manufacturers instructions. For albumin ELISAs, K_3_EDTA‐treated blood and CSF were pre‐diluted 1:4000 and 1:20, respectively, in the provided diluent solution. For sCD163 ELISAs, serum and CSF were both pre‐diluted 1:2 in PBS. Absorbances were measured using a GloMax® Multi Detection System (Promega). A sigmoidal 4PL curve fit (using GraphPad Prism® v6.01) was used for analysis.

### 2.6 Iron quantification in CSF by atomic absorption spectroscopy

Graphite furnace atomic absorption spectroscopy was performed using a Z‐8200 Polarised Zeeman Atomic Absorption Spectrometer (Hitachi). Samples were injected in triplicates in a 15 µL sample volume. Absorbance was measured at 248.3 nm using peak heights for calibration. The limit of detection (LOD) and limit of quantification (LOQ) were calculated according to (34) as 1.05 µg/L and 3.16 µg/L, respectively.

### 2.7 Iron quantification in peripheral tissues by ferrozine‐based assay

Rats were sacrificed by an overdose of CO_2_ and perfused intracardially with 120 mL of PBS. Tissue was homogenised in 5 mM KH_2_PO_4_ buffer (pH 7.2 with NaCl) and protein concentration was determined at 280 nm. Iron content was determined via ferrozine‐based assays (35). Results from FeCl_3_ standard samples were subjected to a linear curve fit and iron concentrations in tissue homogenates were interpolated. The iron content of each sample was related to its protein concentration (determined by absorbance measurements at 280 nm using a NanoDrop (PeqLab)).

### 2.8 Immunohistochemistry

Rats were sacrificed by an overdose of CO_2_ and perfused intracardially with 4% paraformaldehyde (PFA). Brain, spinal cord and peripheral organs were dissected, post‐fixed in 4% PFA for 24 h and routinely embedded in paraffin. Immunohistochemical single stainings via 3,3′‐diaminobenzidine tetrahydrochloride hydrate (DAB) development were performed as described (36). Antigen retrieval for ferroportin was performed by 1 h steaming of tissue sections in 1 mM EDTA/10 mM Tris buffer (pH 8.6); no antigen retrieval was necessary for aquaporin‐1. Ferroportin (NBP2‐75923; Novus Biologicals) and aquaporin‐1 (sc‐20810; Santa Cruz) antibodies were diluted 1:100 and 1:500, respectively.

### 2.9 Turnbull blue staining

DAB‐enhanced Turnbull blue (TBB) staining for the detection of non‐haem iron was performed as described (37). Tissue sections (2–3 µm) were incubated in 2% ammonium sulfide (Merck Millipore) for 1.5 h and subsequently in 20% potassium ferricyanide/1% HCl (both Merck Millipore) for 15 min. Endogenous peroxidase was blocked for 1 h in 0.01 M sodium azide/0.3% H_2_O_2_ in methanol. Finally, tissue sections were incubated in 0.025% DAB/0.005% H_2_O_2_ for 2 h and counterstained with hemalum.

### 2.10 Densitometric quantification

Pictures of the regions of interest (ROI) were taken with standardised acquisition conditions employing NIS software (Leica). Quantification was performed using ImageJ (v1.48); (i) masks were made to exclude unstained background around spinal cord cross‐sections or to exclude parenchymal tissue surrounding choroid plexuses or ependyma and (ii) integrated density was measured within the masks via an automated macro. Data were finally averaged per ROI and rat.

### 2.11 Statistics

For statistical analysis and data representation, GraphPad Prism® v6.01 was used. Bar charts indicate mean ± standard deviation; each individual dot represents a rat. Reported statistics result either from one‐way ANOVAs with Tukey's multiple comparisons tests or unpaired, two‐tailed Student's *t*‐tests. A *p*‐value < 0.05 was considered statistically significant.

## 3 RESULTS

### 3.1 Choroid plexus epithelial cells of LEWzizi rats are strongly iron‐laden

**FIGURE 1:**
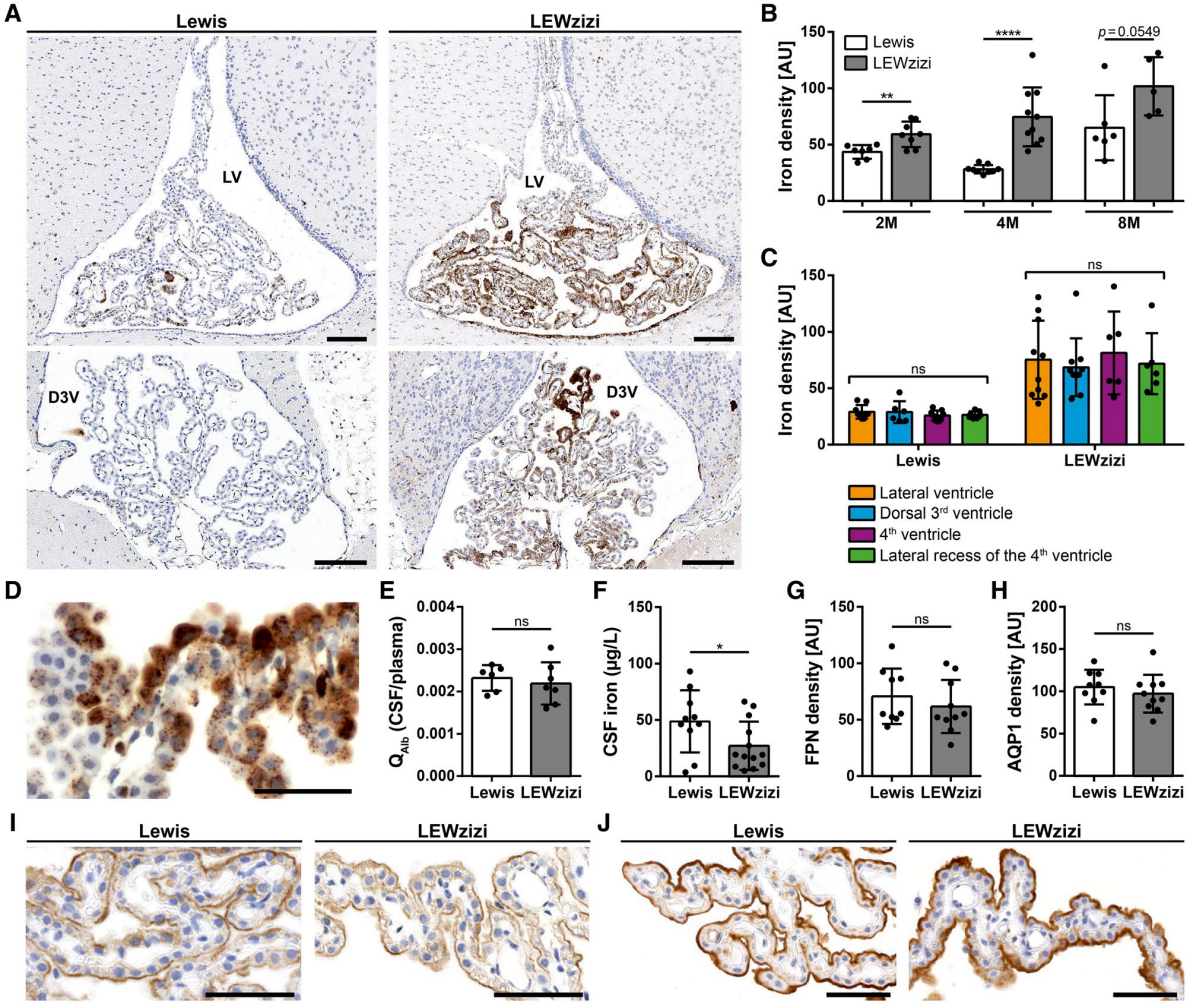
Iron density in the choroid plexus is strongly increased in LEWzizi rats. (A) Iron patterns (TBB staining) in the choroid plexus within the lateral ventricle (LV) and dorsal 3^rd^ ventricle (D3V) of 4‐months‐old (4M) Lewis and LEWzizi rats. Scale bars, 100 µm. (B) Total iron density of choroid plexus. Each data point represents the average of densitometric measurements of iron densities from lateral ventricles, dorsal 3^rd^ ventricles, 4^th^ ventricles and lateral recesses of the 4^t^h ventricle of an individual rat. AU, arbitrary units. (C) Densitometric measurements of iron density in the choroid plexus within the lateral ventricles (orange), dorsal 3^rd^ ventricles (blue), 4^th^ ventricles (magenta) and lateral recesses of the 4^th^ ventricle (green) in 4M Lewis and LEWzizi rats. AU, arbitrary units. (D) Detailed iron patterns of a choroid plexus in the lateral ventricle of a 4M LEWzizi rat. Scale bar, 50 µm. (E) Ratio of albumin concentrations (Q_Alb_) between paired CSF and blood plasma samples taken from 4M Lewis and LEWzizi rats. (F) Iron levels within the cerebrospinal fluid (CSF) of 4M Lewis and LEWzizi rats. (G and H) Densitometric quantification of ferroportin (G; FPN) and aquaporin‐1 (H; AQP1) expression in choroid plexuses within the lateral ventricles of 4M Lewis and LEWzizi rats. AU, arbitrary units. (I and J) Apical expression of ferroportin (I) and aquaporin‐1 (J) in choroid plexus epithelial cells. Representative pictures were taken from choroid plexuses in lateral ventricles of 4M Lewis and LEWzizi rats. Scale bars, 50 µm. (B, E‐H) Reported statistics results from unpaired, two‐tailed Student's *t*‐tests. (C) Reported statistics result from one‐way ANOVAs. (b, c, e‐g) Dots represent individual rats; error bar ± SD; *p < 0.05; **p < 0.01; ****p < 0.0001; ns, not significant

Histochemistry (DAB‐enhanced TBB staining) revealed a strong signal for iron within the choroid plexuses of LEWzizi rats (Figure 1A). Densitometric quantification confirmed that iron densities in the choroid plexuses of 2‐ (2M), 4‐ (4M) and 8‐month‐old (8M) LEWzizi rats were strongly increased compared with age‐matched wild‐type Lewis controls (Figure 1B). In a more detailed analysis, we distinguished between choroid plexuses located within different ventricles (lateral ventricle, dorsal 3^rd^ ventricle, 4^th^ ventricle and lateral recess of the 4^th^ ventricle). Iron densities were not influenced by the localisation of the choroid plexuses at any time point tested (Figure 1C; Figure S1A). For both Lewis and LEWzizi rats, iron is located to choroid plexus epithelial cells, either in a punctuated vesicular or in an intense diffuse staining pattern (Figure 1D). Iron accumulation was also detected in scattered choroid plexus macrophages. Generally, iron distribution within the choroid plexuses was inhomogeneous and patchy (Figure 1A,D). In lateral ventricles, a gradient in choroid plexus iron staining was evident, particularly in LEWzizi rats, with parts proximal to the corpus callosum showing the most intense iron positivity (Figure 1A).

To test for the integrity of the blood–CSF barrier, we determined the albumin concentration ratio between the CSF and peripheral blood. Albumin quotients (Q_Alb_) were similar in Lewis and LEWzizi rats (Figure 1E), indicating an intact blood–CSF barrier interface in LEWzizi rats. Unexpectedly, iron levels in the CSF were significantly decreased in 4M LEWzizi rats compared with age‐matched wild‐type Lewis controls (Figure 1F).

Increased iron densities in the choroid plexuses paired with decreased CSF iron levels could indicate active iron retention in the choroid plexus epithelial cells, e.g. due to decreased expression of the only known iron exporter ferroportin (FPN, SLC40A1). Immunohistochemical stainings showed an apical expression of ferroportin in both Lewis and LEWzizi choroid plexus epithelial cells (Figure 1I). Densitometric quantification confirmed similar expression levels of ferroportin in Lewis and LEWzizi rats (Figure 1G). Similarly, the intense iron loading of choroid plexus epithelial cells in LEWzizi rats neither influenced the apical expression patterns (Figure 1J) nor overall expression levels (Figure 1H) of the main water channel aquaporin‐1, which plays a major role in the production of CSF.

### 3.2 Iron accumulates in the ependymal ventricle walls of LEWzizi rats

**FIGURE 2:**
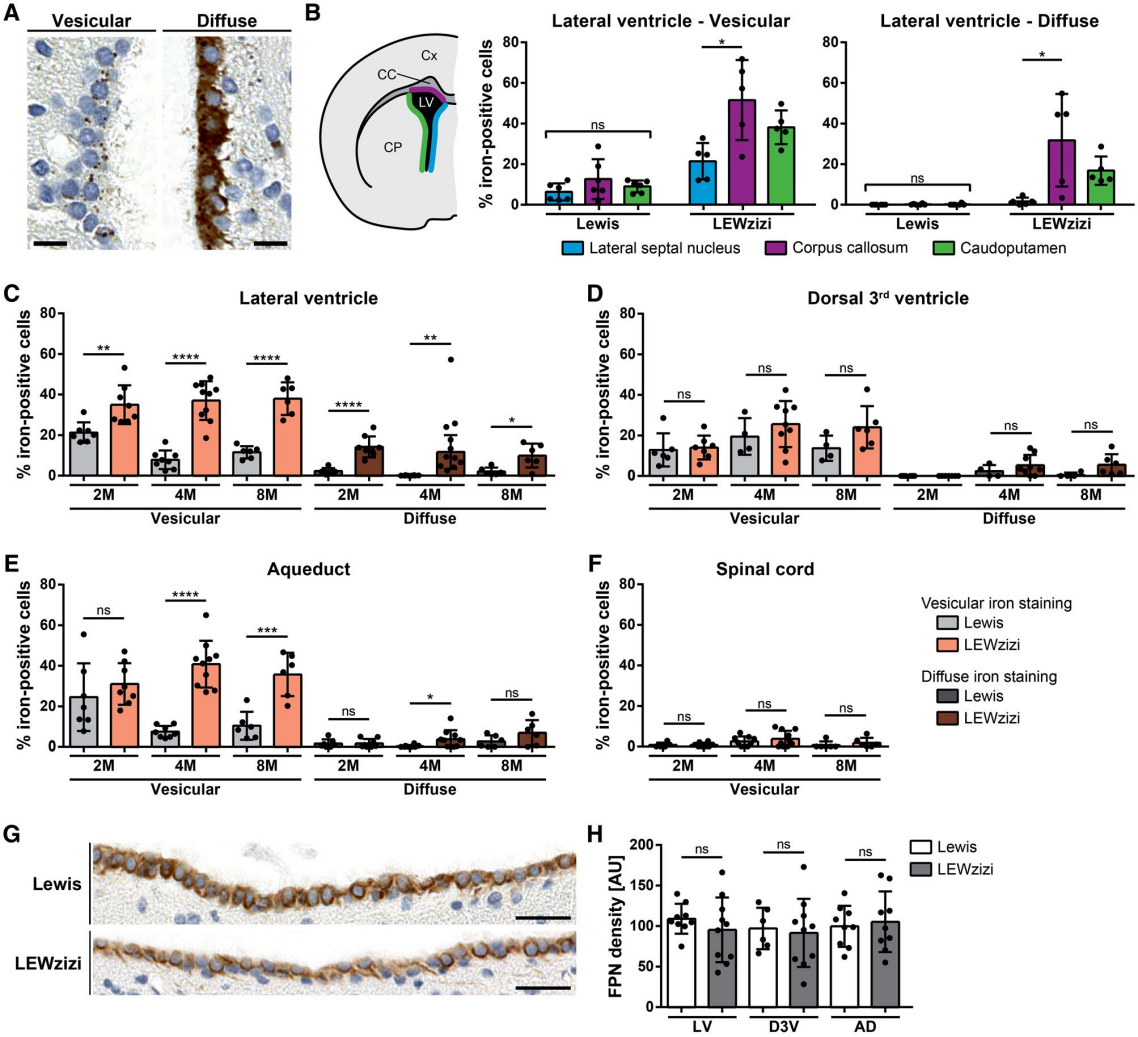
Numbers of iron‐positive ependymal cells are increased in LEWzizi rats. (A) Two major iron patterns were detected in the ependymal ventricular lining: vesicular iron positivity and diffuse cytoplasmic iron accumulation. Representative pictures of DAB‐enhanced TBB stainings were taken from the lateral ventricle of a 4M LEWzizi rat. Scale bars, 10 µm. (B) Percentage of iron‐positive ependymal cells lining different borders of the lateral ventricle of 4M Lewis and LEWzizi rats as shown in the schematic hemispheric cross‐section. Iron staining patterns were categorized as either vesicular or diffuse and were determined separately for ependymal cells proximal to the lateral septal nucleus (blue), corpus callosum (magenta) or caudoputamen/striatum (green). Cx, cortex; CC, corpus callosum; CP, caudoputamen; LV, lateral ventricle. (C‐F) Percentage of iron‐positive ependymal cells lining the lateral ventricle (C), dorsal 3^rd^ ventricle (D), aqueduct (E) or central canal of the spinal cord (F). Data represent the percentage per ventricular compartment independent of the surrounding parenchyma of 2M, 4M and 8M Lewis and LEWzizi rats. Per individual rat and tissue section, between 225 and 1669 (lateral ventricle), 44 and 274 (dorsal 3^rd^ ventricle), 58 and 634 (aqueduct) and 26 and 121 (central canal of the spinal cord) total ependymal cells were counted, of which numbers of iron‐positive cells were registered as well. (G) Predominant basolateral expression of ferroportin in ependymal cells lining the lateral ventricles of 4M Lewis and LEWzizi rats. Scale bars, 25 µm. (H) Densitometric quantification of ferroportin (FPN) expression in the ependymal linings of the lateral ventricles (LV), dorsal 3^rd^ ventricle (D3V) and aqueduct (AD) of 4M Lewis and LEWzizi rats. AU, arbitrary units. (B) Reported statistics result from one‐way ANOVAs followed by Tukey's multiple comparisons tests. (C‐F; H) Reported statistics result from unpaired, two‐tailed Student's *t*‐tests. (B, C‐F) Dots represent individual rats; error bar ± SD; *p < 0.05; **p < 0.01; ***p < 0.001; ****p < 0.0001; ns, not significant

Similar to choroid plexus epithelial cells, the ependymal cells of the ventricle walls of LEWzizi rats also accumulated iron in two distinct staining patterns: a vesicular pattern and a diffuse cytoplasmic iron positivity (Figure 2A). For quantification, we determined the percentage of iron‐positive ependymal cells lining the anatomically different ventricles of 2M, 4M and 8M Lewis and LEWzizi rats. The lateral ventricles and the aqueducts displayed higher percentages of iron‐positive ependymal cells in LEWzizi versus Lewis rats for both vesicular and diffuse staining patterns (Figure 2C,E). In contrast, we did not observe such differences in the 3^rd^ ventricles (Figure 2D). The ependymal cells of the central canal were almost completely devoid of iron and no differences were found between Lewis and LEWzizi rats (Figure 2F). Of note, the aqueduct and the central canal, both belonging to the inner ventricular system, lack an adjacent choroid plexus.

We analysed the lateral ventricles in further detail (Figure 2B) and differentiated between ependyma covering the striatum (caudoputamen), the corpus callosum and the lateral septal nuclei towards the brain midline. For 4M Lewis rats, no differences were found. For 4M LEWzizi rats, however, ventricle walls adjacent to the corpus callosum contained significantly more iron‐positive ependymal cells (both vesicular and diffuse) than the other lateral ventricle walls. These patterns were largely preserved in younger (2M) and older (8M) LEWzizi rats as well (Figure S1B).

The iron exporter ferroportin was pronouncedly expressed at basolateral ependymal cell membranes of both Lewis and LEWzizi rats (Figure 2G). Densitometric quantification of the ependymal lining of lateral ventricles, dorsal 3^rd^ ventricles and aqueducts did not reveal any significant difference between Lewis and LEWzizi rats (Figure 2H).

### 3.3 Iron abnormally accumulates in oligodendrocytes, axonal tracts and microglia in the LEWzizi CNS

**FIGURE 3:**
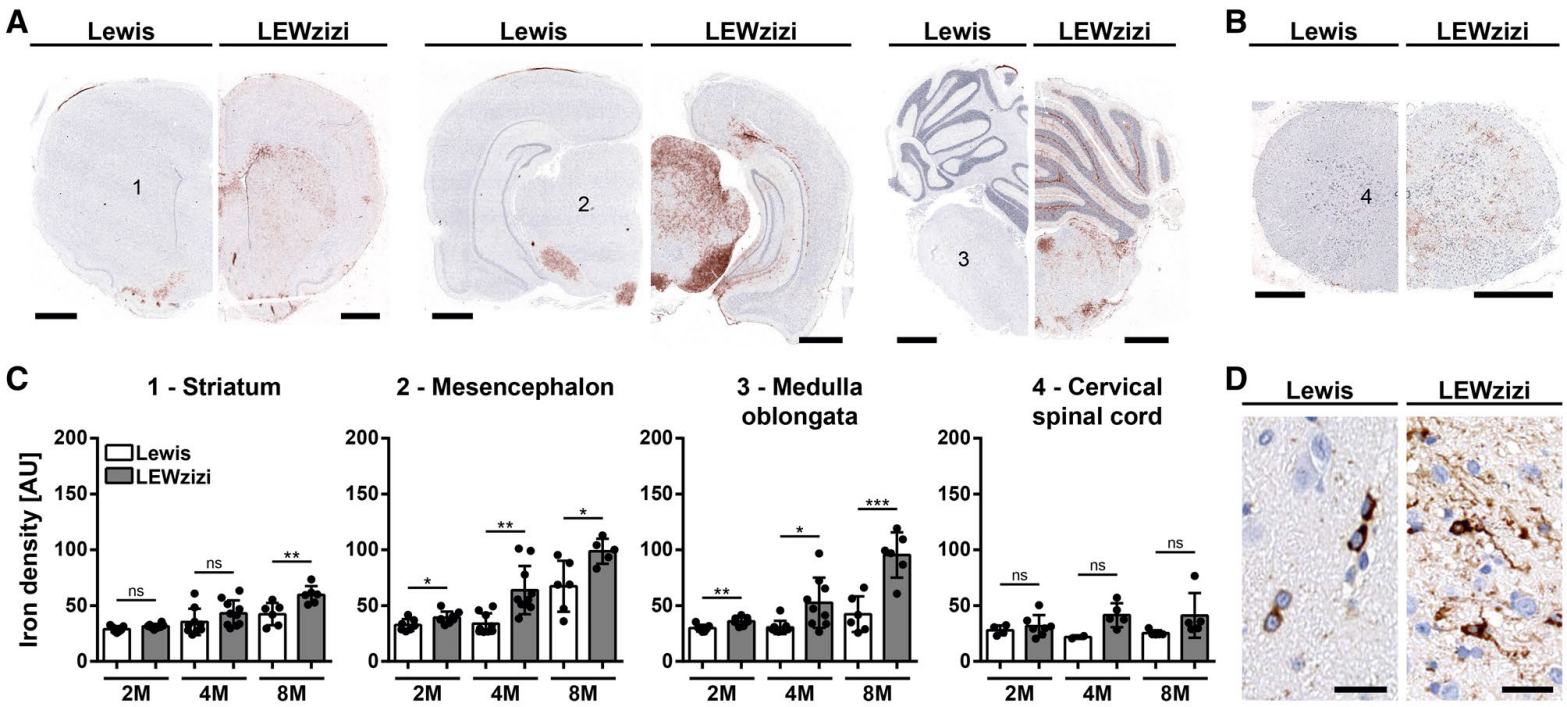
Abnormally high iron accumulation in the brain of LEWzizi rats. (A and B) DAB‐enhanced TBB staining (brown) for the detection of non‐haem iron in representative hemispheric brain (A) and spinal cord (B) cross‐sections of 4‐months‐old (4M) Lewis and LEWzizi rats. Sections were counterstained for nuclei (blue). Numbers refer to regions of interest quantified in (C). (A) Scale bar, 1 mm; (B) scale bar, 500 µm. (C) Densitometric measurements of iron density in the striatum, mesencephalon, medulla oblongata and cervical spinal cord of 2M, 4M and 8M Lewis and LEWzizi rats. Numbers refer to areas of interest in (A), where pictures were taken under standardised conditions. Reported statistics result from unpaired, two‐tailed Student's *t*‐tests. Dots represent individual rats; error bar ± SD; AU, arbitrary units; *p < 0.05; **p < 0.01; ***p < 0.001; ns, not significant. (D) Iron patterns visualised by TBB staining in the mesencephalon of 4M Lewis and LEWzizi rats. Scale bar, 20 µm

Apart from choroid plexuses and ependyma, iron accumulated in the CNS parenchyma of LEWzizi rats. As we have shown previously, iron levels in ageing LEWzizi rats increase more pronouncedly compared with age‐matched wild‐type Lewis rats (32). Here, we deepen the topographical analyses of CNS iron accumulation (Figure 3A–C). The age‐related iron increase in the LEWzizi CNS was particularly pronounced in the mesencephalon, pons, medulla oblongata and cerebellum, and less so in the basal ganglia (e.g. striatum), thalamus and hypothalamus (data of the latter two not shown). Iron levels in the LEWzizi spinal cord increased during ageing compared with Lewis rats, too, but the comparison between genotypes did not reach statistical significance. On the cellular level, we observed strongly pronounced iron accumulation in the LEWzizi brain in axonal tracts and oligodendrocytes, which were to lesser extents also iron‐positive in Lewis rats (Figure 3D). As shown previously, microglia in LEWzizi but not wild‐type Lewis rat brains were strongly iron‐laden (32).

### 3.4 Red blood cells from LEWzizi rats are macrocytic and more fragile

**FIGURE 4:**
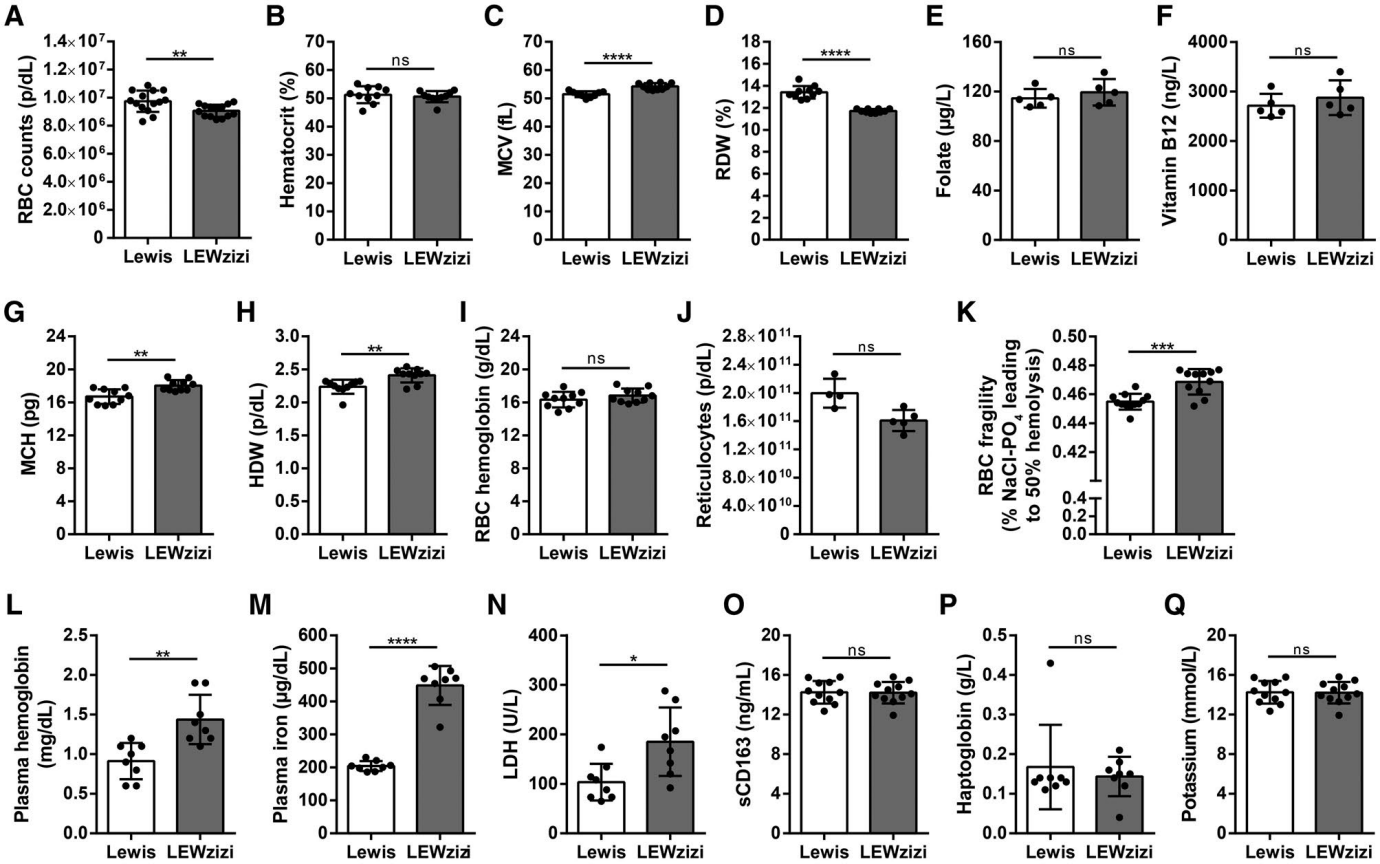
Low‐grade intravascular haemolysis of fragile macrocytic red blood cells in LEWzizi rats. (A‐D, G‐I) Complete blood counts of K_3_EDTA‐treated blood of 4M Lewis and LEWzizi rats. RBC, red blood cell; MCV, mean cell volume of RBCs; RDW, RBC distribution width; MCH, mean cellular haemoglobin content of RBCs; HDW, haemoglobin distribution width. (E and F) Folate (E) and vitamin B12 (F) levels in the serum of 4M Lewis and LEWzizi rats. (J) Reticulocyte counts in K_3_EDTA‐treated blood of 4M Lewis and LEWzizi rats. (K) Osmotic fragility of RBCs upon hypotonic challenge. Depicted is the NaCl concentration leading to 50% haemolysis of RBCs within a blood sample. (L‐N, P, Q) Automated measurements of free haemoglobin (L), cell‐free iron (M), lactate dehydrogenase (LDH, N), haptoglobin (P) and potassium (Q) levels in the plasma of 4M Lewis and LEWzizi rats. (O) Soluble CD163 (sCD163) levels in the serum of 4M Lewis and LEWzizi rats determined by ELISA. Reported statistics result from unpaired, two‐tailed Student's *t*‐tests. Dots represent individual rats; error bar ± SD; *p < 0.05; **p < 0.01; ***p < 0.001; ****p < 0.0001; ns, not significant

Iron is delivered to the choroid plexus and the CNS parenchyma via the blood. To examine if LEWzizi rats show altered iron homeostasis in the peripheral circulation as well, we performed complete blood counts (CBCs). Several major red blood cell (RBC) indices were significantly altered between wild‐type Lewis and LEWzizi rats. RBC counts in 4M LEWzizi rats were slightly but statistically significantly decreased (Figure 4A), while the haematocrit remained unchanged (Figure 4B). LEWzizi RBCs were macrocytic, i.e. their mean cell volume (MCV) was increased (Figure 4C). Moreover, the range of RBC volumes, measured by the RBC distribution width (RDW), was significantly decreased (Figure 4D). The enlarged LEWzizi RBCs were present despite normal folate and vitamin B12 serum levels (Figure 4E,F) and contained higher amounts of haemoglobin per RBC (Figure 4G), which varied to a significantly higher extent as measured by the haemoglobin distribution width (HDW) (Figure 4H). The increase in RBC volume and haemoglobin content fully compensated for the decreased RBC numbers, since total haemoglobin blood concentrations did not differ between Lewis and LEWzizi rats (Figure 4I). We did not observe any statistically significant change in RBC production in the bone marrow, since reticulocyte counts did not differ between genotypes (Figure 4J). Thus, the overall oxygen transport capacity was preserved in LEWzizi rats, thus not fulfilling the core criterion for anaemia. Importantly, LEWzizi RBCs showed significantly increased osmotic fragility upon hypotonic challenge in vitro (Figure 4K).

### 3.5 LEWzizi rats show low‐grade intravascular haemolysis

Both the increased size and the elevated osmotic fragility of LEWzizi RBCs rendered them more susceptible to intravascular haemolysis. Accordingly, cell‐free haemoglobin and iron levels were significantly increased in the plasma of 4M LEWzizi rats (Figure 4L,M). Lactate dehydrogenase (LDH) levels were markedly and significantly elevated in 4M LEWzizi plasma (Figure 4N) suggesting haemolysis. Due to its high pro‐oxidative capacity, cell‐free iron‐laden haemoglobin, supposedly liberated from RBCs during haemolysis, is instantly bound by plasma haptoglobin and effectively cleared by the binding of haptoglobin–haemoglobin complexes to their cell surface receptor CD163 on blood monocytes. Levels of soluble CD163 (sCD163), arising from the shedding of membrane‐bound CD163 via ADAM17 (38), were similar in wild‐type Lewis and LEWzizi serum (Figure 4O). Haptoglobin plasma levels were minimally and non‐significantly reduced in LEWzizi rats (Figure 4P) pointing towards a merely low‐grade intravascular haemolysis of LEWzizi RBCs. Importantly, artificially induced extravascular haemolysis could be excluded due to unchanged plasma potassium levels in LEWzizi rats (Figure 4Q). Our data indicate that the excess cell‐free iron and haemoglobin in the LEWzizi plasma, derived from low‐grade intravascular haemolysis of fragile macrocytic RBCs, are not increasingly scavenged by blood monocytes.

### 3.6 Abnormal iron accumulation in kidney proximal tubules of LEWzizi rats

**FIGURE 5:**
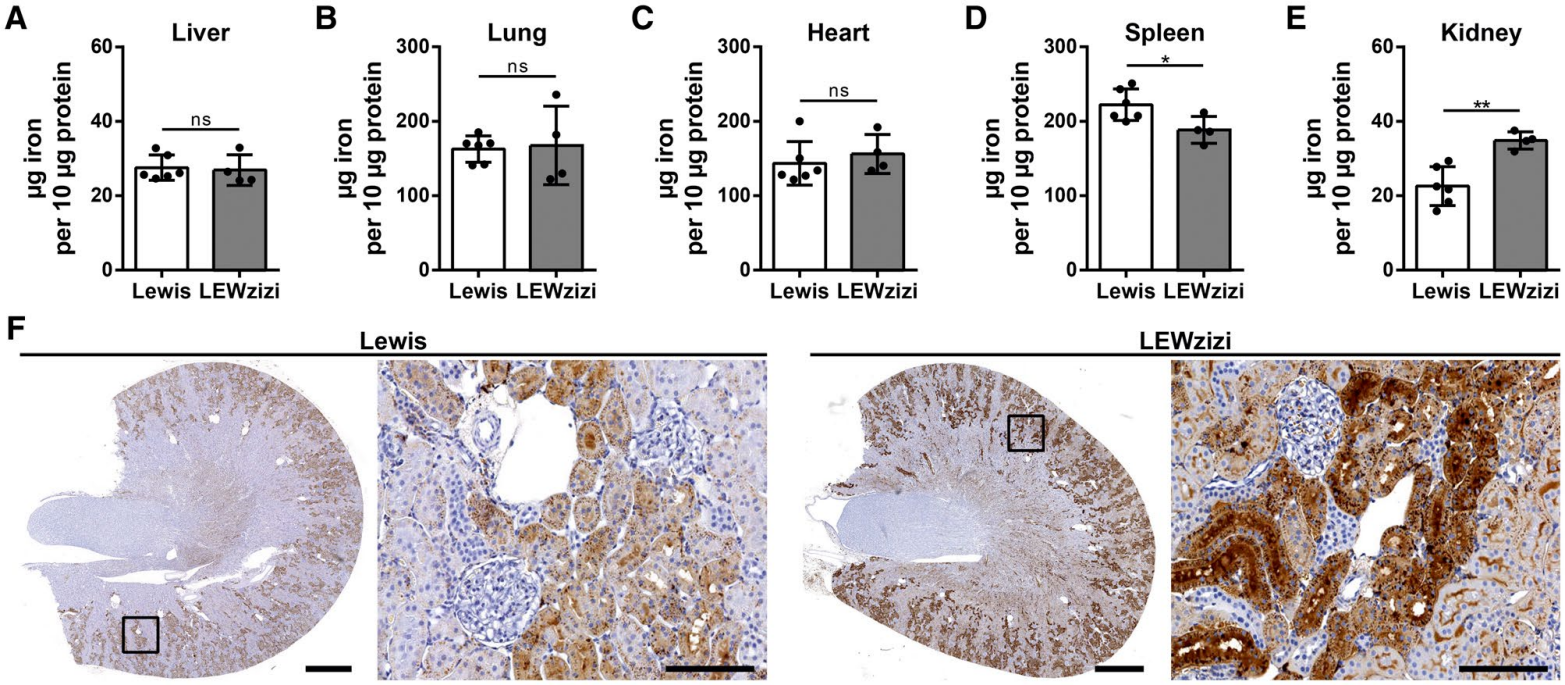
Kidney proximal tubules show increased iron accumulation in LEWzizi rats. (A‐E) Iron quantification via the ferrozene method of liver (A), lung (B), heart (C), spleen (D) and kidney (E) homogenates derived from 4M Lewis and LEWzizi rats. Animals were thoroughly perfused with PBS prior to tissue dissection. Reported statistics result from unpaired, two‐tailed Student's *t*‐tests. Dots represent individual rats; error bar ± SD; *p < 0.05; **p < 0.01; ns, not significant. (F) Iron staining patterns in kidneys of 4M Lewis and LEWzizi rats. Boxed areas in the cross‐sections are shown in higher magnification as well as depicting iron accumulation in renal proximal tubules. Scale bars cross‐sections, 1 mm; scale bars magnification, 100 µm

Despite the significant increases of cell‐free iron in the blood and of cellular iron in the CNS of LEWzizi rats, most peripheral organs such as liver, lung, heart and spleen did not accumulate iron (Figure 5A–D). In contrast, iron levels within the spleen of 4M LEWzizi rats were significantly decreased compared with wild‐type Lewis rats (Figure 5D). These findings underline that cell‐free haemoglobin and iron in the peripheral blood are not increasingly scavenged by the haemoglobin–haptoglobin–CD163 axis, which would lead to elevated disposal of iron in the liver and spleen. Interestingly, kidneys of 4M LEWzizi rats contained significantly elevated amounts of iron (Figure 5E), which is located mostly to proximal tubules (Figure 5F) in the kidney cortex. This observation is of particular interest, since the barrier between blood and kidney proximal tubules shares several similarities with the blood–choroid plexus barrier (39, 40).

## 4 DISCUSSION

Experimental rodent models have proven as valuable tools for mimicking and studying diseases of the human CNS. These models can be further improved, when human age‐related physiological changes and comorbidities are co‐modelled as well. One of these age‐related factors constitutes the accumulation of iron in the CNS, which is pronounced in humans but naturally sparse in rodents. The role of iron as an amplifier of tissue damage has gained increasing interest in various neurological diseases (41); however, the translatability of experimental rodent data to neuroinflammation and neurodegeneration in the human CNS is limited (29). In the current work, we characterised iron accumulation in the brain of LEWzizi rats, which descend from zitter (zi/zi) rats (30, 31) and present with abnormally high iron levels in the CNS (32). There, iron accumulates with age mainly in oligodendrocytes and myelin sheaths in a similar anatomical and cellular distribution as observed in aged humans (29, 32). Here, we further expand our observations to choroid plexuses, ependyma, cerebrospinal fluid, blood and peripheral organs. We report on iron overload within the choroid plexuses and ependymal ventricle walls of LEWzizi rats. Moreover, we found a co‐occurrence between (i) increased iron loads in the LEWzizi CNS and (ii) low‐grade haemolysis with increased plasma levels of cell‐free haemoglobin and iron due to elevated RBC fragility in the peripheral blood of LEWzizi rats. Our data suggest a tight connection between the peripheral blood and the CNS regarding their iron content.

Accumulating data indicate that CNS diseases, as well as normal aging, derange the morphology and functionality of choroid plexus epithelial cells (42, 43, 44, 45), placing choroid plexuses more and more in the focus of studies. Several human diseases of diverse aetiologies have been by now connected to altered iron accumulation in choroid plexuses, such as β‐thalassemia, fragile X‐associated tremor/ataxia syndrome (FXTAS), haemochromatosis and alcoholism (11, 12, 13, 14, 15, 16). Also, the repeated therapeutic administration of RBC concentrates was related to a so‐called “choroid plexus iron overload sign” in magnetic resonance imaging (27, 28). Physiologically, iron within the choroid plexus accumulates most pronouncedly within choroid plexus epithelial cells (46, 47, 48, 49), which accordingly express a variety of molecules for iron import, storage and export (21, 50). In particular, in situ hybridisation of mouse choroid plexus revealed strong mRNA expression of the major iron importer DMT1, the so far only known iron exporter ferroportin and the two ferroxidase ceruloplasmin and hephaestin (21). The ferrireductase DCYTB was apically expressed on choroid plexus epithelial cells, suggesting that they import iron from the CSF via DMT1 and its coupled DCYTB (21); conversely, the expression of the transferrin receptor (TfR1) had a basolateral pattern (22) and was increased in iron deficiency (51). Consistent with other reports (22), we could demonstrate an apical expression of ferroportin in choroid plexus epithelial cells of wild‐type Lewis and LEWzizi rats. In the human disease FXTAS, iron abnormally accumulates in the choroid plexus (22) and certain regions of the CNS, particularly in the striatum and putamen (52), similarly to LEWzizi rats. In FXTAS, choroid plexuses additionally expressed lower levels of ferroportin (22) suggestive of active iron retention by the choroid plexuses. Despite increased iron levels in choroid plexus epithelial cells and decreased iron levels in the CSF of LEWzizi rats, the expression of ferroportin was neither up‐ nor downregulated, indicating that the choroid plexus iron overload was not due to decreased iron export. CSF iron levels have not yet been reported in studies involving human FXTAS patients.

Choroidal and extrachoroidal CSF production/drainage, at least in experimental conditions, depend on aquaporin‐1 and aquaporin‐4 water channels, respectively (53, 54). Here, we studied the expression patterns and intensities of aquaporin‐1 in choroid plexuses as a read‐out for putative alterations in choroid plexus functionality and CSF production. However, we did not detect any abnormalities that might point towards altered CSF turnover and thus explain significantly decreased iron levels in the CSF of LEWzizi rats. Despite developmental and transcriptional heterogeneity between the choroid plexuses located in the different cavities of the ventricular system (55, 56, 57, 58, 59), we did not observe divergent iron densities, aquaporin‐1 or ferroportin expression between the lateral, 3^rd^ and 4^th^ ventricles. However, we noticed gradients and hot spots of iron accumulation within each individual choroid plexus. Particularly in the lateral ventricles towards sites close to the corpus callosum, choroid plexus epithelial cells were most intensely packed with iron. This pattern was not reiterated by ferroportin expression. It was, however, spatially mirrored by the iron loading of the opposite ependymal lining of the ventricles. Ependymal cells form a partial flow barrier for solutes between ventricles and the CNS parenchyma (60). They highly express the iron importer DMT1, which follows the strong expression of the iron regulatory proteins IRP1 and IRP2 and indicates their propensity towards iron accumulation upon increased iron availability in surrounding tissue or fluids (61). Taken together, the spatially correlating iron accumulation within ependymal cells and choroid plexus epithelial cells together with decreased CSF iron levels suggest active sequestration and buffering of iron in these barriers in order to reduce the potential toxicity of iron overload in the CNS. Also, it is tempting to speculate that the high density of oligodendrocytes in the corpus callosum and thus a higher continuous demand of iron in this area might be responsible for this particular pattern, which was also present in wild‐type Lewis rats, although to a lesser degree.

Analysis of the peripheral blood revealed increased RBC fragility and decreased RBC numbers for LEWzizi rats compared with wild‐type rats. Unaltered blood reticulocyte counts and therefore unaltered RBC production indicate maintained oxygen transport capacity in the LEWzizi blood. LEWzizi rats were thus not anaemic, likely since RBC decrease was compensated by the observed macrocytosis, i.e. increased RBC volume and increased haemoglobin per RBC. RBC volumes in zitter rats were already previously reported to be significantly increased in comparison with control rats; however, in that study, the RBC haemoglobin content was significantly decreased (62). Since LEWzizi haptoglobin plasma levels were not decreased in our study, we interpret and summarise our findings as a low‐grade but not manifest intravascular haemolysis. Various causes may render RBCs more fragile and susceptible to intravascular haemolysis, like bacterial or parasitic infections and some inherited diseases. Additionally, alterations in lipid homeostasis may affect physical cell membrane properties potentially influencing RBC size and fragility. Homozygous zitter (zi/zi) rats, the ancestors of LEWzizi rats, do present with such alterations. Blood serum levels of total cholesterol, phospholipids and high‐density lipoprotein were significantly lower in zitter (zi/zi) rats compared with Sprague Dawley control rats (62). In brain tissue of zitter (zi/zi) rats, a significant reduction of cholesterol and ganglioside levels was reported (63). Whole‐genome microarray studies using LEWzizi spinal cords [supplementary data in (32)] have shown that the most significantly altered pathways included cholesterol biosynthesis, metabolism of lipids and lipoproteins, sphingolipid metabolism and regulation of phospholipid catabolic processes. These metabolic alterations may very well affect RBC membrane properties and thus explain our observations regarding LEWzizi RBC stability and fragility. We suggest that increased RBC fragility and concomitant intravascular haemolysis with haemoglobin iron liberation are a plausible source of increased plasma and ensuing choroid plexus and CNS iron in the LEWzizi rat. A similar constellation, i.e. concomitant changes in plasma/RBC lipids and iron blood parameters, was reported for the inflammatory demyelinating disease multiple sclerosis (MS). The composition of plasma lipid species was altered and cholesterol levels were significantly reduced in RBCs from MS patients (64). Moreover, abnormally increased diameters of RBCs from MS patients (65, 66), an elevated RBC fragility towards osmotic and mechanical stress (67, 68, 69, 70, 71) and elevated free serum haemoglobin levels have been reported (72).

Despite increased iron and cell‐free haemoglobin levels in the blood, peripheral tissues of LEWzizi rats were mostly spared from iron accumulation, except for the cortex of the kidneys, where a spatially confined accumulation of iron in proximal tubule epithelial cells was observed. The kidney proximal tubule epithelium constitutes a barrier between blood and urine, which resembles the blood–CSF barrier of the choroid plexus in many aspects. Common denominators are fluid transport rates, driving force, expression of transporters to expel exogenous substances and the expression of the principal transepithelial water channel aquaporin‐1. However, the net fluid transport direction across kidney proximal tubules is opposite from that across choroid plexuses epithelial cells [epithelial barriers reviewed in (39, 40, 73)]. Thus, our findings highlight that peripheral organs such as kidneys should never be overlooked particularly in studies involving barrier interfaces.

Our study has some limitations. First, the observational character of our study does not allow causal inferences regarding the observed haemolysis and choroid plexus/ependymal iron accumulation. Particularly the largely unknown functions of attractin should be considered when interpreting our data. Second, age is an important determinant of iron accumulation in the CNS. We cannot rule out that the iron accumulation observed in LEWzizi rats are merely exaggerated ageing effects due to LEWzizi co‐morbidities such as spongiform degeneration. The latter fact also restricted the age range in our study and the oldest animals we could include were 8 months old. Nevertheless, our study adds to the still insufficient data on mechanisms and consequences of iron overload in the choroid plexus and ventricular system in the CNS. Our data highlight the importance of choroid plexuses in iron homeostasis and its role as a putative entry site for iron into the CNS. We also extend the previous characterisation of iron accumulation in the CNS of LEWzizi rats (32) with data on increased iron loads in choroid plexus epithelial cells and ependymal cells of the ventricle lining. We also found evidence for macrocytic red blood cells, which were more fragile due to osmotic challenge, leading to low‐grade haemolysis and significantly increased levels of iron and cell‐free haemoglobin in the peripheral blood, which is to the best of our knowledge the first report of such a co‐occurrence in an animal model.

## CONFLICT OF INTEREST

SHa received speaker's honoraria from Biogen, Sanofi Aventis and unrestricted research grant from Merck. HL received honoraria for lectures from Novartis, Biogen and Sanofi Aventis. Moreover, he is a member of advisory boards at Roche and Medday. TB received personal fees from pharmaceutical companies marketing drugs for multiple sclerosis. The other authors declare no competing financial or non‐financial interests specific to this study.

## AUTHOR CONTRIBUTIONS

IW designed and performed experiments and analysed data. IW and SHa interpreted data. CS initiated the project and performed experiments. TK, SHi and BSM performed or helped with experiments. SU and MB provided substantial materials and knowledge. HL and SHa supervised the study. IW and SHa wrote the manuscript; TB and HL contributed to manuscript editing. All authors read and approved the final manuscript and agree with its content.

## ETHICAL APPROVAL

Experimental work was approved by the Ethics Committee of the Medical University Vienna and performed with the licence of the Austrian Ministry for Science and Research.

## Funding information

This study was supported by the Austrian Science Fund (FWF; projects P27744‐B27, P24245‐B19, UE10207001 and APW1205‐B09). Moreover, TK received a scholarship for academic research support from MSD K.K

## Supporting information


**FIGURE S1** Iron in the choroid plexus and ependymal ventricle linings. (A) Densitometric measurements of iron densities in the lateral ventricles (orange), dorsal 3rd ventricles (blue), 4th ventricles (magenta) and lateral recesses of the 4th ventricle (green) in 2‐months‐old (2M) and 8‐months‐old (8M) Lewis and LEWzizi rats. (B) Percentage of iron‐positive ependymal cells lining different borders of lateral ventricle of 2M and 8M Lewis and LEWzizi rats. Iron staining patterns were categorised as either vesicular or diffuse and were determined separately for ependymal cells proximal to the lateral septal nucleus (blue), corpus callosum (magenta) or caudoputamen/striatum (green). (A) Reported statistics result from one‐way ANOVAs. (B) Reported statistics result from unpaired, two‐tailed Student's *t*‐tests. (A and B) Dots represent individual rats; error bar ± SD; *p < 0.05; **p < 0.01; ***p < 0.001; ****p < 0.0001; ns, not significant

## Data Availability

The data that support the findings of this study are available from the corresponding author upon reasonable request.
