# Supplementary material for: Iron accumulation in the choroid plexus, ependymal cells and CNS parenchyma in a rat strain with low‐grade haemolysis of fragile macrocytic red blood cells
Source: Brain Pathol. 2021 Jan 29;31(2):333–45. doi: 10.1111/bpa.12920 (PMC8018038; doi:10.1111/bpa.12920)
Supplement: Supplementary file 1 — FIGURE S1 Iron in the choroid plexus and ependymal ventricle linings. (A) Densitometric measurements of iron densities in the lateral ventricles (orange), dorsal 3rd ventricles (blue), 4th ventricles (magenta) and lateral recesses of the 4th ventricle (green) in 2‐months‐old (2M) and 8‐months‐old (8M) Lewis and LEWzizi rats. (B) Percentage of iron‐positive ependymal cells lining different borders of lateral ventricle of 2M and 8M Lewis and LEWzizi rats. Iron staining patterns were categorised as either vesicular or diffuse and were determined separately for ependymal cells proximal to the lateral septal nucleus (blue), corpus callosum (magenta) or caudoputamen/striatum (green). (A) Reported statistics result from one‐way ANOVAs. (B) Reported statistics result from unpaired, two‐tailed Student's t‐tests. (A and B) Dots represent individual rats; error bar ± SD; *p < 0.05; **p < 0.01; ***p < 0.001; ****p < 0.0001; ns, not significant [file BPA-31-333-s001.pdf]

Supplementary Data

Iron accumulation in the choroid plexus, ependymal cells and CNS parenchyma in a rat strain with low-grade haemolysis of fragile macrocytic red blood cells

Isabella Wimmer[1,2], Cornelia Scharler[2], Taro Kadowaki[2,3], Sophie Hillebrand[2], Barbara Scheiber-Mojdehkar[4], Shuichi Ueda[5], Monika Bradl[2], Thomas Berger[1], Hans Lassmann[2], Simon Hametner[2,6]

[1] Department of Neurology, Medical University of Vienna, Austria
[2] Department of Neuroimmunology, Center for Brain Research, Medical University of Vienna, Austria
[3] Department of Neurology, Dokkyo Medical University, Tochigi, Japan
[4] Department of Medical Chemistry and Pathobiochemistry, Medical University of Vienna, Austria
[5] Department of Histology and Neurobiology, Dokkyo Medical University, Tochigi, Japan
[6] Division of Neuropathology and Neurochemistry, Department of Neurology, Medical University of Vienna, Austria

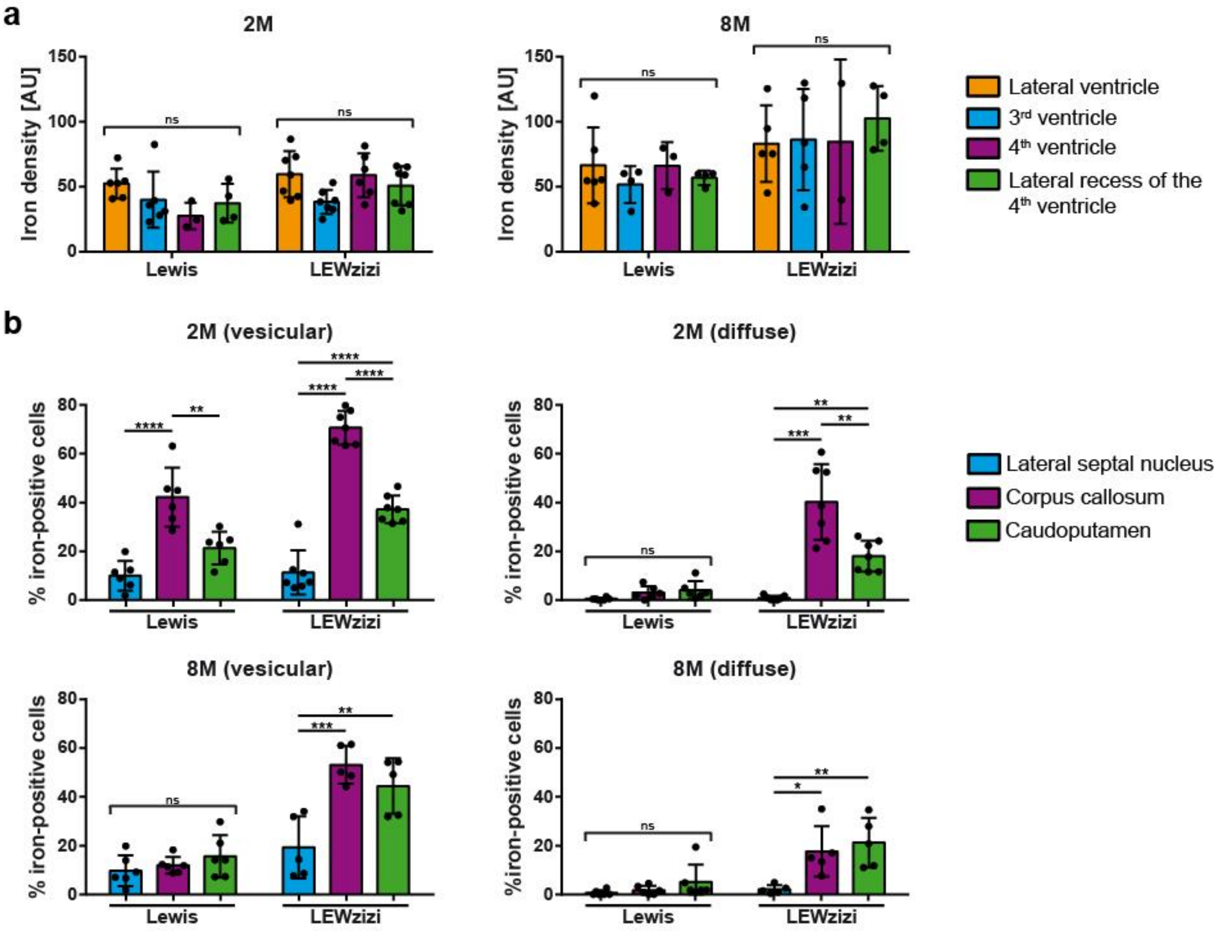

Supplementary Figure 1: Iron in the choroid plexus and ependymal ventricle linings

(a) Densitometric measurements of iron densities in the lateral ventricles (orange), dorsal 3rd ventricles (blue), 4th ventricles (magenta) and lateral recesses of the 4th ventricle (green) in 2-months-old (2M) and 8-months-old (8M) Lewis and LEWzizi rats.

(b) Percentage of iron-positive ependymal cells lining different borders of lateral ventricle of 2M and 8M Lewis and LEWzizi rats. Iron staining patterns were categorised as either vesicular or diffuse and were determined separately for ependymal cells proximal to the lateral septal nucleus (blue), corpus callosum (magenta) or caudoputamen/striatum (green).

(a) Reported statistics result from one-way ANOVAs. (b) Reported statistics result from unpaired, two-tailed Student's t-tests. (a, b) Dots represent individual rats; error bar ± SD; *, $p < 0.05$; **, $p < 0.01$; ***, $p < 0.001$; ****, $p < 0.0001$; ns, not significant
